# Supplementary material for: The Role of Galanin during Bacterial Infection in Larval Zebrafish
Source: Cells. 2021 Aug 6;10(8):2011. doi: 10.3390/cells10082011 (PMC8391356; doi:10.3390/cells10082011)
Supplement: Supplementary file 1 [file cells-10-02011-s001.zip › Figure S1.pdf]

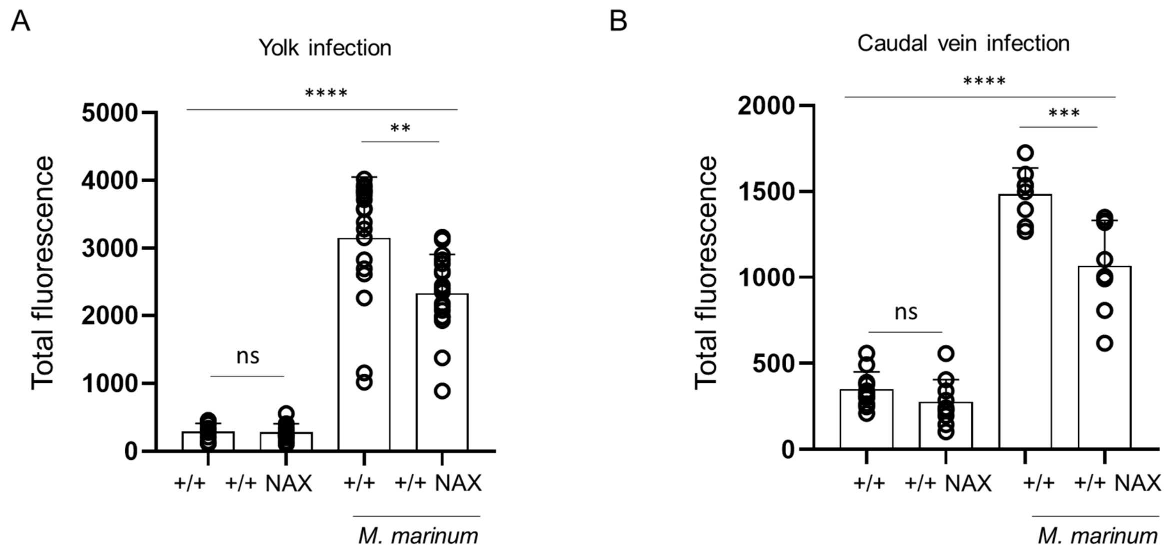

Figure S1. (**A**)(**B**) Bacterial burden in non-infected wild-type larvae, infected wild-type larvae and, infected and non-infected, wild-type larvae treated with NAX 5055 after (**A**) *M. marinum* yolk or (**B**) caudal vein infection. Data is combined from three biological replicates. **P<0.01; ***P<0.001; ****P<0.0001; ns, non-significant.
