# Supplementary material for: The Role of Galanin during Bacterial Infection in Larval Zebrafish
Source: Cells. 2021 Aug 6;10(8):2011. doi: 10.3390/cells10082011 (PMC8391356; doi:10.3390/cells10082011)
Supplement: Supplementary file 1 [file cells-10-02011-s001.zip › Figure S2.pdf]

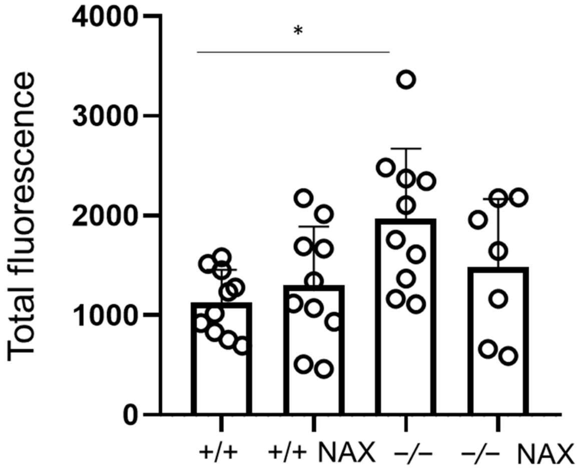

Figure S2. Quantification of macrophages in the *gal*+/+ Tg(*mpeg1*:EGFP) (+/+) and *gal*−/− Tg(*mpeg1*:EGFP) zebrafish larvae. Total fluorescence of *gal*+/+ Tg(*mpeg1*:EGFP) (+/+) and *gal*−/− Tg(*mpeg1*:EGFP) (−/−) without or with (+/+ NAX; −/− NAX) NAX 5055 treatment. Data is combined from three biological replicates (n=10 larvae/group).*$P<0.05$
