# Supplementary material for: The Role of Galanin during Bacterial Infection in Larval Zebrafish
Source: Cells. 2021 Aug 6;10(8):2011. doi: 10.3390/cells10082011 (PMC8391356; doi:10.3390/cells10082011)
Supplement: Supplementary file 1 [file cells-10-02011-s001.zip › Table S1.pdf]

GENE	Forward 5′-3′	Reverse 5′-3′	Accession
irg1l	GGTTAGAAGCAAGTCCTC	TGTGTTCATCCTCCTCAG	NM_001077607
il1b	GAACAGAATGAAGCACATCAAACC	ACGGCACTGAATCCACCAC	NM_212844
tnfa	AGACCTTAGACTGGAGAGATGAC	CAAAGACACCTGGCTGTAGAC	NM_212829
cxcl8a	TGTGTAATTGTTTTCTGGCATTTC	GCGACAGCGTGGATCTACAG	XM_001342570
ppial	ACACTGAAACACGGAGGCAAAG	CATCCACAACCTTCCCGAACAC	AY391451

Table S1. Primer sequences and Genbank accessions for genes analyzed in this study.
