# Supplementary material for: The Role of Galanin during Bacterial Infection in Larval Zebrafish
Source: Cells. 2021 Aug 6;10(8):2011. doi: 10.3390/cells10082011 (PMC8391356; doi:10.3390/cells10082011)
Supplement: Supplementary file 1 [file cells-10-02011-s001.zip › Table S2.pdf]

Galanin receptor expression in *gal*+/+ after *M. marinum* infection

**Ensembl gene ID**	Abbreviated gene name	Fold change	P-value
ENSDARG00000005522	galr1a	-1.075620697	0.829210
ENSDARG00000079165	galr1b	-1.047225932	0.825372
ENSDARG00000020853	galr2a	1.053631371	0.908397
ENSDARG00000105113	galr2b	1.086749172	0.276419

Galanin receptor expression in *gal*−/− after *M. marinum* infection

**Ensembl gene ID**	Abbreviated gene name	Fold change	P-value
ENSDARG00000005522	galr1a	1.134369372	0.68301
ENSDARG00000079165	galr1b	-1.038377255	0.88766
ENSDARG00000020853	galr2a	1.78971366	0.13027
ENSDARG00000105113	galr2b	1.034847303	0.64456

Galanin receptor expression in *gal*−/−

**Ensembl gene ID**	Abbreviated gene name	Fold change	P-value
ENSDARG00000005522	galr1a	-1.029615704	0.93750
ENSDARG00000079165	galr1b	1.331773648	0.17923
ENSDARG00000020853	galr2a	2.012035741	0.07667
ENSDARG00000105113	galr2b	1.049433167	0.64678

Table S2. Expression of galanin receptors in *gal*+/+ and *gal*−/− after *M. marinum* infection and *gal*−/− without infection.
